# The complete chloroplast genome sequence of *Ficus sarmentosa* (Moraceae, Rosales), a widely distributed fig tree in East Asia

**DOI:** 10.1080/23802359.2022.2115860

**Published:** 2022-09-05

**Authors:** Zhen Zhang, De-Shun Zhang

**Affiliations:** College of Architecture and Urban Planning, Tongji University, Shanghai, China

**Keywords:** *Ficus*, plastid genome, climbing fig, phylogenomics

## Abstract

*Ficus sarmentosa* is a common climbing fig tree in East Asia in Moraceae, and its particular geographical distributed pattern and on-going radiation evolution make it significant to explore evolutionary history and biogeography of *Ficus*. In this work, the first complete chloroplast genome of *F. sarmentosa* was reported using Illumina NovaSeq high-throughput sequencing data. Totally, the whole chloroplast genome of *F. sarmentosa* is 160,183 bp in length, which includes large single-copy region of 88,307 bp, small single-copy region of 20,080 bp, and two pairs of inverted repeat regions of 25,898 bp. In addition, GC content and microsatellite makers of the genome were explored. Lastly, phylogenetic analysis demonstrated the closest relationship between *F. sarmentosa* and *F. pumila*.

*Ficus* Linnaeus is the largest genus in Moraceae with extreme species diversity as well as ecologically and evolutionarily important on account of unique obligate mutualism to fig wasp, providing an ideal case for studies of speciation and co-evolution (Wang et al. 2021). Nevertheless, *Ficus* species that have available complete chloroplast genomes in open data repository such as GenBank are less than five percent, rendering that molecular data in this species-rich genus are now far insufficient to support the further researches. *Ficus sarmentosa* Buch.-Ham. ex J.E.Sm. 1810 is a widely distributed climbing fig tree throughout East Asia but unexpectedly absent in adjacent Southeast Asia region (Zhou and Gilbert 2003), in view of the latter is thought to be the distributed center and possible ancestral area of *Ficus* (Cruaud et al. 2012; Pederneiras et al. 2018). Additionally, *F. sarmentosa* possesses up to eight indistinguishable varieties and has numerous allies in subsect. *Plagiostigma*, which forms a species complex under rapid radiation evolution (Zhang et al. 2020). Therefore, studies of its genetic characteristics, including the chloroplast genome and phylogenetic analysis, are of the essence.

In the study, the sample of *F. sarmentosa* was obtained from Putuo Mountain in Zhoushan District, Zhejiang, China (122.40 E, 30.02 N) and the voucher specimen (collector: Lu Zou and Hong-Qing Li, collected number: ZZ063) was deposited in the herbarium of East China Normal University (HSNU, Curator: Rui-Liang Zhu, email: rlzhu@bio.ecnu.edu.cn). Total genomic DNA was extracted from dried leaves using CTAB method (Doyle and Doyle 1987). The shotgun library was prepared based on the total genomic DNA after shearing, end repair and adapter ligation. 2G clean paired-end data (deposited in SRA with the accession number SRR16894930) were obtained with Illumina NovaSeq PE150 platform (San Diego, CA). The chloroplast genome was assembled using GetOrangelle v1.7.5 (Jin et al. 2020) and annotated via PGA v1.0 (Qu et al. 2019) under default parameters. The complete chloroplast genome sequence was deposited in GenBank (accession number: OL415083).

**Figure 1.**
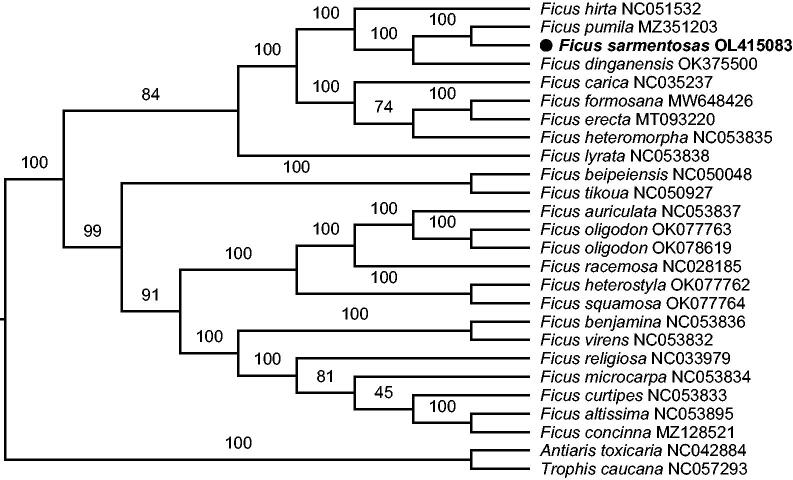
The maximum-likelihood phylogenetic tree based on complete chloroplast genomes. Numbers above the branches indicated the values from 10,000 ultrafast bootstrap.

As a result, the complete genome sequence of *F. sarmentosa* is 160,183 bp in length, consisting of a large single-copy (LSC) of 88,307 bp, a small single-copy (SSC) of 20,080 bp, and two inverted repeats (IRs) of 25,898 bp. In addition, the overall GC content is 35.99%. Generally, 131 genes were annotated, which included 86 coding genes, 37 tRNA genes, and eight ribosomal RNA genes. What is more, 46 microsatellite loci with mononucleotide repeat unit, eight with dinucleotide unit and one with trinucleotide unit, in total 55 microsatellite loci were detected throughout the chloroplast genome via MISA-web (https://webblast.ipk-gatersleben.de/misa/) under default parameters, and these genetic resources are meaningful to the exploration of population genetics and evolutionary history in *F. sarmentosa* species complex. To investigate the phylogenetic placement of *F. sarmentosa*, the maximum-likelihood (ML) tree was reconstructed with 23 available chloroplast genomes of *Ficus* in GenBank via IQ-tree v2.1.3 (Nguyen et al. 2015) under 10,000 ultrafast bootstrap (Hoang et al. 2018), after aligned by MAFFT v7.490 (Katoh and Standley 2013). The related species *Antiaris toxicaria* (Pers.) Lesch. 1810 and *Trophis caucana* (Pittier) C.C.Berg 1988 were selected as outgroup. The ML tree showed that *F. sarmentosa* is close to *F. pumila* Linnaeus 1753 (Figure 1), which is a species of the same subsection, consistent with pervious results (Zhang et al. 2020). The complete chloroplast genome of the widely distributed species *F. sarmentosa* will be promising for a better understanding of population structure, evolutionary history, and biogeography in *Ficus*.

## Data Availability

The genome sequence data that support the findings of this study are openly available in GenBank of NCBI at https://www.ncbi.nlm.nih.gov under the accession no. OL415083. The associated BioProject, SRA, and Bio-Sample numbers are PRJNA771926, SRR16894930, and SAMN23012429, respectively.
